# A barrier island perspective on species–area relationships

**DOI:** 10.1002/ece3.4726

**Published:** 2018-12-08

**Authors:** Christoph Scherber, Hagen Andert, Rolf Niedringhaus, Teja Tscharntke

**Affiliations:** ^1^ Department of Crop Science, Agroecology University of Göttingen Göttingen Germany; ^2^ Institute of Landscape Ecology University of Münster Muenster Germany; ^3^ Landscape Ecology Group, Institute of Biology and Environmental Sciences Carl von Ossietzky University of Oldenburg Oldenburg Germany; ^4^ Centre of Biodiversity and Sustainable Land Use (CBL) Göttingen Germany

**Keywords:** habitat diversity, invertebrates, island ontogeny, nature conservation, plants, sedimentation, species richness of vertebrates

## Abstract

Predictions of species richness by island area are a classical cornerstone in ecology, while the specific features of barrier islands have been little appreciated. Many shorelines are occupied by barrier islands, which are shaped by offshore sedimentation processes and annual storm tide events. Hence, the appearance of these islands may vary between years if they are not protected by dykes. Here, we analyzed more than 2,990 species across 36 taxonomic groups (including vertebrates, invertebrates, and land plants) on German barrier islands, the East Frisian Islands. We tested for relationships between species richness or species incidence and island area (SAR), island habitat diversity and further island parameters using a range of generalized linear and mixed‐effects models. Overall species richness was explained best by habitat diversity (Shannon index of habitat types). Analyses on the occurrence probability of individual species showed that changes of barrier island area by sedimentation and erosion, that is, barrier island‐specific dynamics, explained the occurrence of 17 of 34 taxa, including most beetles, plants, and birds. Only six taxa such as spiders (249 species) and mammals (27 species) were primarily related to area. The diversity of habitat types was a key predictor for the incidence of twenty‐five taxa, including ground beetles, true bugs and grasshoppers, amphibians, and reptiles. Overall, richness and incidence of taxa differed greatly in their responses, with area (although varying from 0.1 to 38.9 km^2^) playing a minor and island heterogeneity a major role, while barrier island‐specific sedimentation and erosion turned out to additionally explain species richness and occurrence.

## 1 INTRODUCTION

Analyses of species–area relationships have stimulated the understanding of key ecological and evolutionary processes such as immigration‐extinction dynamics (MacArthur & Wilson, 2001). According to the equilibrium theory of island biogeography (MacArthur & Wilson, 2001), island area and geographical isolation are the major factors predicting the islands’ species numbers. Along with these factors, habitat diversity has emerged as another important variable influencing species richness of oceanic islands (Rosenzweig, 1995). Island biogeographical models have been reviewed by several authors (Heaney, 2000; Losos, Ricklefs, & MacArthur, 2010), who showed that factors such as island age, disturbance history, but also species traits may affect species richness on islands. Environmental and physical predictors have recently also been shown to be important for the prediction of plant biodiversity in 17,883 islands worldwide (Weigelt, Jetz, & Kreft, 2013).

So far, oceanic islands have been the main study objects of island biogeography. While these islands are geologically stable and often distant from the mainland, there are also islands that consist of rather mobile sediments and occur closer to the shorelines. About 2,149 of these so‐called barrier islands (Hayes, 2005) occupy c. 20,783 km of worldwide shorelines. Almost 20 percent of worldwide barrier islands extend along the coastline of the Atlantic Ocean and the Gulf of Mexico (Stutz & Pilkey, 2011).

The East Frisian Islands (Germany) form a chain of barrier islands along the mid‐European coastline of the North Sea. These islands never had contact to the mainland and have also not been formed around mainland cores. Due to their independence from mainland, they offer an opportunity to study species distribution of flora and fauna along a longitudinal coast line gradient (all islands oriented as a string of islands).

**Figure 1:**
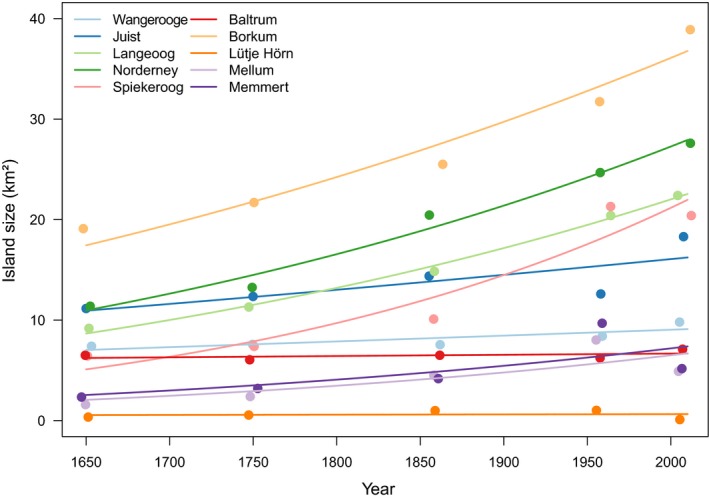
Trajectories of island size for each East Frisian Island from 1650 to 2008 (excluding the artificial island of Minsener Oog). Note that Lütje Hörn showed a small decline in island growth between 1960 and 2000, as it had been nearly destroyed by a tidal storm event in 1976. All other islands increased in size over the 400‐year period

In this study, we consider ten barrier islands for which we compiled an unprecedented dataset on biodiversity, habitat types, and several centuries of expansion processes. Processes of island growth have been described from 1650 onwards (Homeier, Stephan, & Niemeyer, 2010; Niedringhaus, Haeseler, & Janiesch, 2008; Petersen & Pott, 2005; Figure 1). Former studies on species–area relationships often analyzed responses of only a few taxa and only few island characteristics (Franzen, Schweiger, & Betzholtz, 2012; Kier et al., 2009; MacArthur & Wilson, 2001), whereas we included 34 taxa and a wealth of island features into our approach.

We analyze data on overall richness of taxa (multidiversity sensu Allan et al., 2014) and taxonomic composition (using multinomial models). In addition, we employ binomial incidence functions for all species within a taxon, that is, we directly model the occurrence probability of each taxon for each island.

Assuming that ontogenetic island data should be important for the richness and occurrence of taxa, we hypothesize:

1. Current species richness and incidence of barrier islands is significantly influenced by ontogenetic island data such as annual sedimentation rates.
2. Habitat diversity of islands calculated by Shannon index has a greater impact on species richness and incidence than area.

## 2 MATERIAL AND METHODS

### 2.1 Study area

The study area is located at the coastal line of the Northwestern state of Lower Saxony (Germany, 53°35′17″N, 6°40′11″E–53°43′16″N, 8°8′58″E). This chain of barrier islands consists of eleven islands, of which ten islands have their natural origin exclusively from both sedimentation and erosion processes and annually occurring storm tides (Homeier et al., 2010; Petersen & Pott, 2005; Streif, 1990). Island formation started about 4,000 years ago (atlantic to subboreal part of the Holocene epoch). Extreme disturbances mainly driven by storms and intertidal changes formed a unique landscape with large colonies of breeding sea birds and seals in the Northern Sea, which belongs to the UNESCO World Heritage Site “Lower Saxon Wadden Sea National Park” since 1993.

### 2.2 Island physiognomy

For each island, we collected data on island‐specific habitat types (e.g., grasslands, dykes, dunes, salt marshes, fresh water habitats) and the island size from 2008 from Niedringhaus et al., 2008). Island size varied from 0.1 to 38.9 km^2^ (Niedringhaus et al., 2008). We calculated the Shannon index of habitat types (based on each habitat´s area in hectares; Niedringhaus et al., 2008), retrieved the island sizes for the years of 1650, 1750, 1860, 1960, and 2008 from historical maps (Homeier et al., 2010; Niedringhaus et al., 2008) and calculated different aspects of island ontogeny (Table 1). We measured the increase in island area over time and the minimal and maximal size of each island (described below). This allowed us to calculate the coefficient of variation (CV) of the different island sizes, the differences in island size (delta), and the maximal erosion and sedimentation rate of island size changes as indicators of island disturbances (referred to as “erosion” and “sedimentation”). We used the R package “nlme” in combination with a self‐starting power model to fit the increase in annual island size via the formula b*year^c^, where “b” is the intercept and c the slope of island growth (referred as the parameter “island growth”). All variables are described in Table 1.

**Table 1:** Overview of explanatory variables with their ranges and units. Data derived from Niedringhaus et al., 2008; Homeier et al., 2010

Abbreviation	Variable	Minimum	Maximum	Unit
Area	Area in 2008	0.10	38.90	km^2^
Habitat diversity	Shannon index of habitat types per island	1.02	2.44	–
Island growth	Slope of linear regression model with area data from 1650 to 2008	0.35	7.41	–
CV	Coefficient of variation of island area between 1650 and 2008	6.14	66.51	km^2^
Erosion	Maximal amount of erosion since 1650	−25.50	−0.45	km^2^
Sedimentation	Maximal amount of sedimentation since 1650	0.65	27.99	km^2^
Delta	Sedimentation minus erosion	1.12	53.49	km^2^

Note

CV, coefficient of variation.

### 2.3 Plant and animal biodiversity data

In 2009, the Wadden Sea National Park authorities published a summary of island biodiversity across a wide range of taxa, dating from the early 20th century up to now (Niedringhaus et al., 2008). This dataset documents presence or absence data of more than 9,000 species with about 6,926 extant species. In the present analysis, we consider species recorded for the period after 1976. We classified taxa into 34 taxonomic groups from lichens up to mammals of 10 East Frisian Islands. The dataset of the island Minsener Oog had to be excluded as this island had been artificially built as a tidal power plant and therefore had been artificially planted and colonized. Further details can be found in Niedringhaus et al. (2008). For statistical reasons, we considered only taxa that were present on at least three islands. This resulted in 5,444 species used in further analyses.

### 2.4 Statistical analyses

Data analysis was split into three parts: (a) analysis of overall richness of taxa for each island; (b) analysis of taxonomic composition (multinomial models) for each island; and (c) analysis of the occurrence of individual species within each taxon per island (species incidence).

Data were analyzed using R 3.5.1 on 64‐bit architecture (R Development Core Team, 2018) running in an RStudio environment (RStudio Team, 2016). We started off with the following list of potential explanatory variables (Table 1, Supporting Information Tables S1 and S2): island changes between 1650 and 2008 (sedimentation, erosion, delta, increase, CV), habitat diversity, and island area.

To analyze overall species richness, we calculated multidiversity as the summed species richness (sum of the occurrences) of all taxa (Allan et al., 2014) and entered each of the seven explanatory variables into separate models that were then compared using AICc (Burnham & Anderson, 2002). To analyze taxonomic composition, we set up three different multinomial models (for plants, vertebrates, and invertebrates) with the same explanatory variables that were again compared using AICc. Finally, to analyze incidence of species within each taxon, a broader range of statistical models was then set up using all possible pairwise combinations of these explanatory variables, but excluding those that were highly correlated (erosion and sedimentation; growth in island size and erosion; growth in island size and sedimentation; heterogeneity and area; cv and heterogeneity; see Supporting Information Table S1). This resulted in a set of 23 statistical models (see Supporting Information Table S2).

We then set up separate generalized linear mixed‐effects models (R package lme4, version 1.1‐18‐1, Bates, Maechler, Bolker, & Walker, 2015) for each taxon with binomial presence/absence of each individual taxon as the response variable, and island as a random effect. Individual species were not added as random effects, as this would have saturated the models with random effects. An observation‐level random effect (to account for potential overdispersion) was not justified as AICc‐values increased when including it. Models were fitted using a local, derivative‐free optimization algorithm (BOBYQUA; Powell, 2009). Fixed‐effects terms were all pairwise combinations of explanatory variables, excluding those that were highly correlated (|*r*| > 0.6) (Table 2, Table S2).

**Table 2:** Parameter estimates from minimal adequate generalized linear mixed‐effects models on binomial presence/absence of species within each taxon

Taxon	Variable	Intercept	Habitat diversity	Delta (log)	CV	Increase	Area	Erosion (log)	Sedimentation (log)
*Invertebrates*									
Apidae	Estimate	−6.09	3.07	–	–	–	–	–	–
Apidae	*SE*	1.12	0.55	–	–	–	–	–	–
Apidae	*z*‐value	−5.45	5.59	–	–	–	–	–	–
Apidae	Pr(>|*z*|)	0.00	0.00	–	–	–	–	–	–
Coleoptera: Hydradephaga, Palpicornia	Estimate	−6.93	2.86	0.44	–	–	–	–	–
Coleoptera: Hydradephaga, Palpicornia	*SE*	0.87	0.40	0.17	–	–	–	–	–
Coleoptera: Hydradephaga, Palpicornia	*z*‐value	−7.96	7.16	2.53	–	–	–	–	–
Coleoptera: Hydradephaga, Palpicornia	Pr(>|*z*|)	0.00	0.00	0.01	–	–	–	–	–
Araneae	Estimate	−0.71	–	–	–	–	0.08	−0.54	–
Araneae	*SE*	0.42	–	–	–	–	0.02	0.28	–
Araneae	*z*‐value	−1.71	–	–	–	–	4.06	−1.94	–
Araneae	Pr(>|*z*|)	0.09	–	–	–	–	0.00	0.05	–
Auchenorrhyncha	Estimate	−4.43	2.36	–	–	–	–	–	–
Auchenorrhyncha	*SE*	0.66	0.33	–	–	–	–	–	–
Auchenorrhyncha	*z*‐value	−6.71	7.19	–	–	–	–	–	–
Auchenorrhyncha	Pr(>|*z*|)	0.00	0.00	–	–	–	–	–	–
Blattodea	Estimate	0.40	–	–	−0.04	–	–	–	–
Blattodea	*SE*	1.01	–	–	0.03	–	–	–	–
Blattodea	*z*‐value	0.40	–	–	−1.33	–	–	–	–
Blattodea	Pr(>|*z*|)	0.69	–	–	0.18	–	–	–	–
Brachycera	Estimate	−5.60	–	0.83	–	0.51	–	–	–
Brachycera	*SE*	1.07	–	0.38	–	0.14	–	–	–
Brachycera	*z*‐value	−5.25	–	2.16	–	3.61	–	–	–
Brachycera	Pr(>|*z*|)	0.00	–	0.03	–	0.00	–	–	–
Crustacea	Estimate	−5.05	2.10	–	–	–	–	–	–
Crustacea	*SE*	2.20	1.09	–	–	–	–	–	–
Crustacea	*z*‐value	−2.29	1.93	–	–	–	–	–	–
Crustacea	Pr(>|*z*|)	0.02	0.05	–	–	–	–	–	–
Hymenoptera: Formicidae	Estimate	−4.19	2.55	–	–	–	–	–	–
Hymenoptera: Formicidae	*SE*	1.05	0.53	–	–	–	–	–	–
Hymenoptera: Formicidae	*z*‐value	−3.98	4.81	–	–	–	–	–	–
Hymenoptera: Formicidae	Pr(>|*z*|)	0.00	0.00	–	–	–	–	–	–
Gastropoda	Estimate	0.48	–	–	−0.06	–	–	–	–
Gastropoda	*SE*	0.55	–	–	0.01	–	–	–	–
Gastropoda	*z*‐value	0.86	–	–	−4.20	–	–	–	–
Gastropoda	Pr(>|*z*|)	0.39	–	–	0.00	–	–	–	–
Coleoptera: Carabidae	Estimate	−3.27	1.20	–	–	0.16	–	–	–
Coleoptera: Carabidae	*SE*	0.56	0.28	–	–	0.05	–	–	–
Coleoptera: Carabidae	*z*‐value	−5.79	4.35	–	–	3.20	–	–	–
Coleoptera: Carabidae	Pr(>|*z*|)	0.00	0.00	–	–	0.00	–	–	–
Heteroptera	Estimate	−4.66	1.98	–	–	–	–	0.44	–
Heteroptera	*SE*	0.64	0.32	–	–	–	–	0.17	–
Heteroptera	*z*‐value	−7.29	6.16	–	–	–	–	2.57	–
Heteroptera	Pr(>|*z*|)	0.00	0.00	–	–	–	–	0.01	–
Lepidoptera	Estimate	−8.23	3.04	–	–	–	–	–	–
Lepidoptera	*SE*	3.25	1.60	–	–	–	–	–	–
Lepidoptera	*z*‐value	−2.53	1.90	–	–	–	–	–	–
Lepidoptera	Pr(>|*z*|)	0.01	0.06	–	–	–	–	–	–
Acari	Estimate	−7.58	2.48	–	–	–	–	–	–
Acari	*SE*	3.58	1.73	–	–	–	–	–	–
Acari	*z*‐value	−2.12	1.44	–	–	–	–	–	–
Acari	Pr(>|*z*|)	0.03	0.15	–	–	–	–	–	–
Odonata	Estimate	−6.96	3.27	–	–	–	–	–	–
Odonata	*SE*	2.42	1.18	–	–	–	–	–	–
Odonata	*z*‐value	−2.88	2.78	–	–	–	–	–	–
Odonata	Pr(>|*z*|)	0.00	0.01	–	–	–	–	–	–
Coleoptera (others)	Estimate	−8.65	3.12	–	–	0.37	–	–	–
Coleoptera (others)	*SE*	1.41	0.65	–	–	0.11	–	–	–
Coleoptera (others)	*z*‐value	−6.12	4.78	–	–	3.56	–	–	–
Coleoptera (others)	Pr(>|*z*|)	0.00	0.00	–	–	0.00	–	–	–
Coleoptera (phytophagous)	Estimate	−17.86	7.89	–	–	–	–	–	–
Coleoptera (phytophagous)	*SE*	2.92	1.35	–	–	–	–	–	–
Coleoptera (phytophagous)	*z*‐value	−6.12	5.86	–	–	–	–	–	–
Coleoptera (phytophagous)	Pr(>|*z*|)	0.00	0.00	–	–	–	–	–	–
Saltatoria	Estimate	−5.21	2.77	–	–	–	–	–	–
Saltatoria	*SE*	1.22	0.60	–	–	–	–	–	–
Saltatoria	*z*‐value	−4.29	4.63	–	–	–	–	–	–
Saltatoria	Pr(>|*z*|)	0.00	0.00	–	–	–	–	–	–
Siphonaptera	Estimate	−5.13	1.83	–	–	–	–	–	0.71
Siphonaptera	*SE*	1.52	0.71	–	–	–	–	–	0.36
Siphonaptera	*z*‐value	−3.37	2.58	–	–	–	–	–	1.97
Siphonaptera	Pr(>|*z*|)	0.00	0.01	–	–	–	–	–	0.05
Coleoptera: Staphylinidae	Estimate	−2.60	–	–	–	0.36	0.04	–	–
Coleoptera: Staphylinidae	*SE*	0.39	–	–	–	0.08	0.02	–	–
Coleoptera: Staphylinidae	*z*‐value	−6.67	–	–	–	4.27	2.65	–	–
Coleoptera: Staphylinidae	Pr(>|*z*|)	0.00	–	–	–	0.00	0.01	–	–
Hymenoptera: Symphyta	Estimate	−7.42	3.29	–	–	–	–	0.27	–
Hymenoptera: Symphyta	*SE*	0.69	0.32	–	–	–	–	0.14	–
Hymenoptera: Symphyta	*z*‐value	−10.81	10.36	–	–	–	–	1.95	–
Hymenoptera: Symphyta	Pr(>|*z*|)	0.00	0.00	–	–	–	–	0.05	–
Trichoptera	Estimate	−2.71	–	–	–	–	0.07	–	–
Trichoptera	*SE*	0.72	–	–	–	–	0.03	–	–
Trichoptera	*z*‐value	−3.77	–	–	–	–	2.13	–	–
Trichoptera	Pr(>|*z*|)	0.00	–	–	–	–	0.03	–	–
Hymenoptera: Aculeata (excl. Apidae, Formicidae)	Estimate	−6.48	2.88	–	–	–	–	0.40	–
Hymenoptera: Aculeata (non‐Apidae)	*SE*	0.91	0.44	–	–	–	–	0.21	–
Hymenoptera: Aculeata (non‐Apidae)	*z*‐value	−7.08	6.56	–	–	–	–	1.89	–
Hymenoptera: Aculeata (non‐Apidae)	Pr(>|*z*|)	0.00	0.00	–	–	–	–	0.06	–
*Vertebrates*									
Aves	Estimate	−1.64	1.89	–	–	–	–	–	–
Aves	*SE*	1.10	0.56	–	–	–	–	–	–
Aves	*z*‐value	−1.48	3.38	–	–	–	–	–	–
Aves	Pr(>|*z*|)	0.14	0.00	–	–	–	–	–	–
Pisces	Estimate	−3.23	–	–	–	–	0.15	−0.64	–
Pisces	*SE*	0.62	–	–	–	–	0.03	0.30	–
Pisces	*z*‐value	−5.18	–	–	–	–	5.26	−2.10	–
Pisces	Pr(>|*z*|)	0.00	–	–	–	–	0.00	0.04	–
“Reptilia”	Estimate	−5.73	2.39	–	–	–	–	–	–
“Reptilia”	*SE*	3.84	1.79	–	–	–	–	–	–
“Reptilia”	*z*‐value	−1.49	1.33	–	–	–	–	–	–
“Reptilia”	Pr(>|*z*|)	0.14	0.18	–	–	–	–	–	–
Lissamphibia	Estimate	−6.53	3.36	–	–	–	–	–	–
Lissamphibia	*SE*	2.83	1.37	–	–	–	–	–	–
Lissamphibia	*z*‐value	−2.31	2.44	–	–	–	–	–	–
Lissamphibia	Pr(>|*z*|)	0.02	0.01	–	–	–	–	–	–
Mammalia	Estimate	−1.15	–	–	−0.03	–	0.10	–	–
Mammalia	*SE*	0.38	–	–	0.01	–	0.02	–	–
Mammalia	*z*‐value	−3.06	–	–	−3.60	–	6.34	–	–
Mammalia	Pr(>|*z*|)	0.00	–	–	0.00	–	0.00	–	–
*Plantae*									
Spermatophyta (herbaceous)	Estimate	−0.18	–	–	−0.03	–	0.07	–	–
Spermatophyta (herbaceous)	*SE*	0.23	–	–	0.00	–	0.01	–	–
Spermatophyta (herbaceous)	*z*‐value	−0.77	–	–	−6.78	–	9.08	–	–
Spermatophyta (herbaceous)	Pr(>|*z*|)	0.44	–	–	0.00	–	0.00	–	–
Spermatophyta (trees)	Estimate	−7.75	3.83	–	–	–	–	–	–
Spermatophyta (trees)	*SE*	0.92	0.44	–	–	–	–	–	–
Spermatophyta (trees)	*z*‐value	−8.44	8.74	–	–	–	–	–	–
Spermatophyta (trees)	Pr(>|*z*|)	0.00	0.00	–	–	–	–	–	–
Monocotyledonae: Poaceae	Estimate	−4.71	2.46	–	–	–	–	–	–
Monocotyledonae: Poaceae	*SE*	0.56	0.28	–	–	–	–	–	–
Monocotyledonae: Poaceae	*z*‐value	−8.36	8.83	–	–	–	–	–	–
Monocotyledonae: Poaceae	Pr(>|*z*|)	0.00	0.00	–	–	–	–	–	–
Spermatophyta (shrubs)	Estimate	−6.19	2.97	–	–	–	–	–	–
Spermatophyta (shrubs)	*SE*	0.81	0.39	–	–	–	–	–	–
Spermatophyta (shrubs)	*z*‐value	−7.68	7.66	–	–	–	–	–	–
Spermatophyta (shrubs)	Pr(>|*z*|)	0.00	0.00	–	–	–	–	–	–
Pteridophyta	Estimate	−6.34	3.12	–	–	–	–	–	–
Pteridophyta	*SE*	1.78	0.85	–	–	–	–	–	–
Pteridophyta	*z*‐value	−3.57	3.65	–	–	–	–	–	–
Pteridophyta	Pr(>|*z*|)	0.00	0.00	–	–	–	–	–	–
Bryophyta	Estimate	−8.75	3.34	–	–	0.22	–	–	–
Bryophyta	*SE*	1.51	0.69	–	–	0.11	–	–	–
Bryophyta	*z*‐value	−5.80	4.84	–	–	2.06	–	–	–
Bryophyta	Pr(>|*z*|)	0.00	0.00	–	–	0.04	–	–	–
*Lichenes*									
Lichenes	Estimate	−24.12	10.30	–	–	–	–	–	–
Lichenes	*SE*	10.04	4.61	–	–	–	–	–	–
Lichenes	*z*‐value	−2.40	2.24	–	–	–	–	–	–
Lichenes	Pr(>|*z*|)	0.02	0.03	–	–	–	–	–	–

Note

CV, coefficient of variation; Pr(>|z|), *p*‐value; SE, standard error of the mean.

For each taxon, we generated a total of 23 models and compared them using Akaike's information criterion, corrected for small sample sizes (AICc). In our final set of best models, we included models whose AICs differed by 2 from the minimum value of AIC (after Burnham & Anderson, 2002) to assess models around the best fitting model (S1). We inspected model residuals for constant variance and normality.

## 3 RESULTS

**Figure 2:**
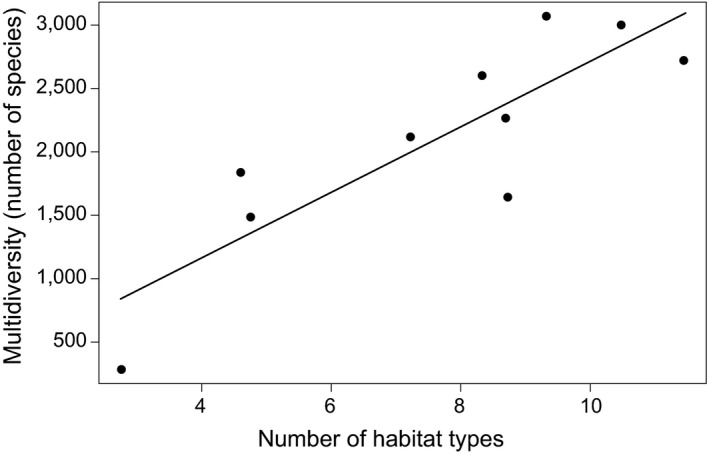
Multidiversity (number of taxa, summed) as a function of the (back‐transformed) number of habitat types per island. Multidiversity was calculated from incidence data; hence, no abundance weighting was possible in this case

Multidiversity was best explained by habitat diversity (AICc = 48.88), followed by island area (AICc = 49.06) and ontogenetic island parameters (AICc > 49.20). There was a strong positive effect of habitat diversity on multidiversity (Figure 2).

Multinomial models showed that taxonomic composition of invertebrates, vertebrates, and plants was also best explained by habitat diversity or ontogenetic island parameters, with island area only playing a minor role: For invertebrates, the coefficient of variation in island size, habitat diversity, and island growth was better predictors than other explanatory variables (AICc = 75,717, 75,780, and 76,182, respectively). For vertebrates, habitat diversity (AICc = 1,219) was slightly better supported by the data than island area (AICc = 1,221). For plants, island growth, CV, and habitat diversity were the best predictors (AICc = 12,317, 12,337 and 12,343, respectively).

**Figure 3:**
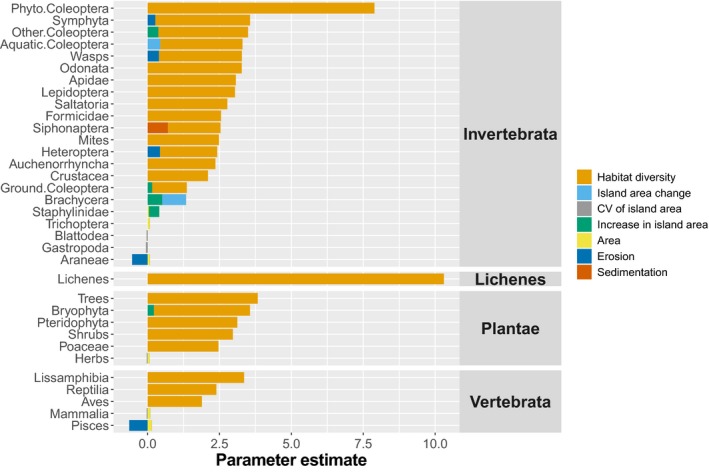
A visual summary of generalized linear mixed‐effects model parameter estimates (on a logit scale), showing that habitat diversity was the major parameter affecting incidence across taxa. Intercepts not shown for clarity (see Table 2 for details). Graphical interpretation of these parameters is best performed by looking at Figures 4 and 5

**Figure 4:**
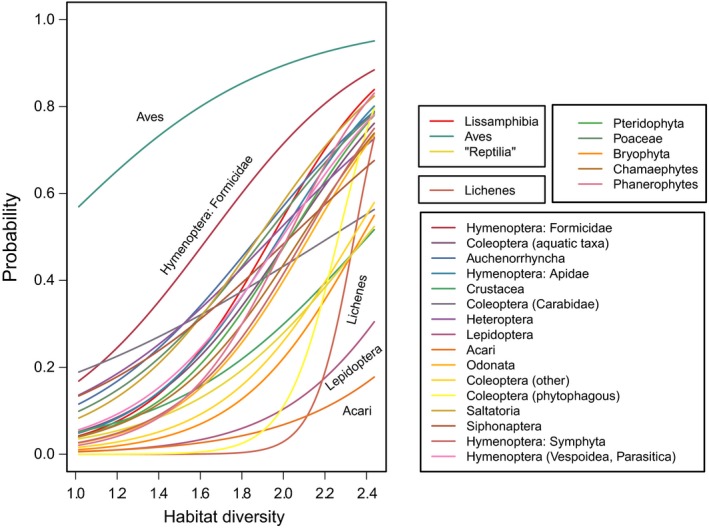
Taxa that responded positively to habitat diversity across the three major taxonomic groups (invertebrates, vertebrates, plants). Lines show predictions from multinomial models

**Figure 5:**
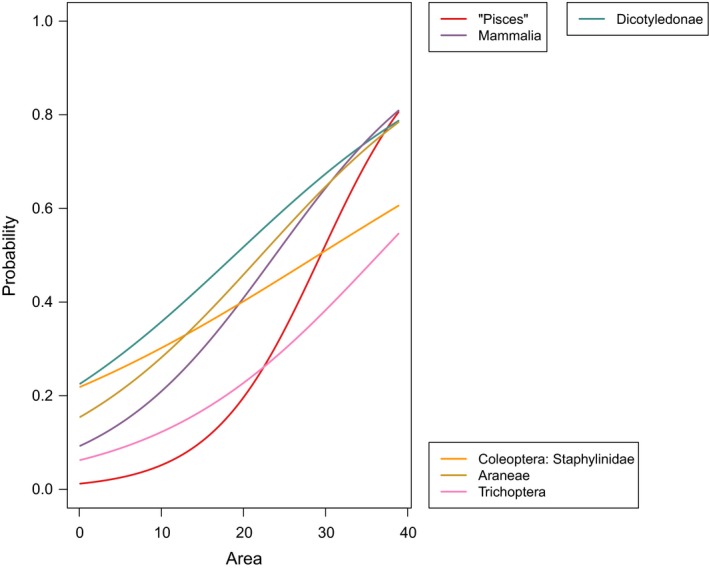
Taxa that responded positively to area (in km^2^) across the major taxonomic groups. Lines show predictions from multinomial models

The analyses of species incidence using binomial generalized linear mixed‐effect models for all taxa (Table 2; Figure 3) showed that habitat diversity explained the occurrence of more than 70% of taxa (25 of 34 taxa; Figure 4), while island area was only selected in about 17% of cases (6 of 34 taxa; Figure 5). Island‐specific parameters explained the occurrence of 50% of taxa (17 of 34). Habitat diversity had nonlinear effects on the occurrence probability of taxa, with most responses showing S‐shaped curves (Figure 4). Birds were a taxon that showed high occurrence probabilities throughout, even at low habitat diversity. By contrast, invertebrate taxa such as Lepidoptera, phytophagous beetles, Acari, and lichens increased only at high habitat diversity (Figure 4). The taxa that responded primarily to area were freshwater fishes and mammals, dicotyledonous plants, and three invertebrate groups (Figure 5).

**Figure 6:**
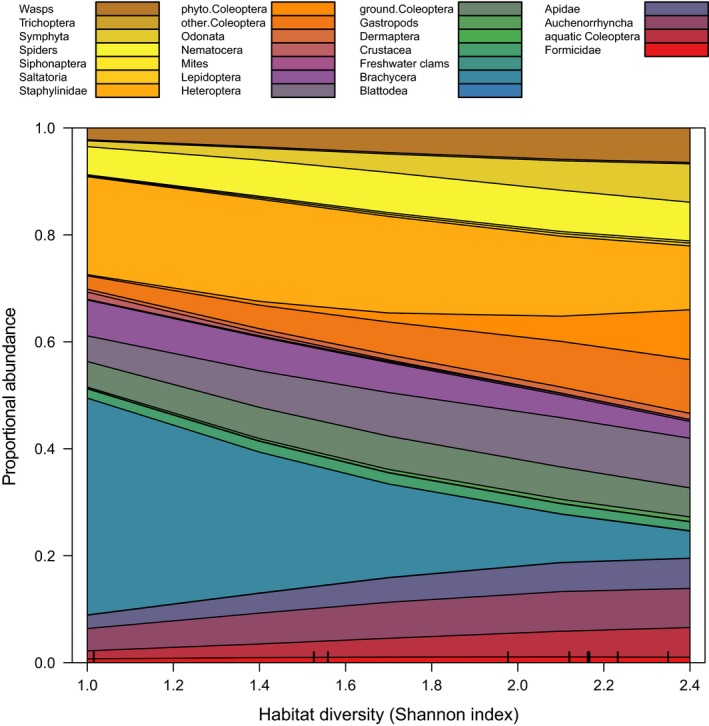
Patterns in taxonomic structure of invertebrate taxa as a function of habitat diversity, inferred from multinomial models. Note the decreasing importance of flies (Diptera: Brachycera) with increasing habitat diversity

Finally, when looking at proportional abundance across major invertebrate taxa (Figure 6), flies (Diptera: Brachycera) turned out to be dominant at low habitat diversity, while other taxa (especially phytophagous beetles) increased in relative abundance at higher habitat diversity.

## 4 DISCUSSION

The East Frisian Islands cover a broad range of distinct habitat types (e.g., old lower salt marshes, coastal dune valleys, swamps) with associated plant communities and a manifold of breeding, hunting, and mating habitats for animals. In the present study, covering 34 taxa from 10 German barrier islands, island area turned out to be much less important for biodiversity than habitat diversity. Habitat diversity was found to be of paramount importance for most taxa. Additionally, island changes caused by decade‐long sedimentation and erosion processes contributed considerably to island biodiversity across taxa.

Whether area per se (island biogeography theory; MacArthur & Wilson, 2001) or habitat heterogeneity (niche theory) affect species diversity has been a long debated issue in ecology (Kadmon & Allouche, 2007; Tews et al., 2004). In the present study, using a wide range of statistical models competing for explanatory power, heterogeneity was the winner in many cases and for many taxa. This is not to say that area was unimportant—rather, our results indicate that area clearly was not the most parsimonious explanatory variable. Instead, a likely conclusion is that both area and habitat heterogeneity are needed for high biodiversity on barrier islands. In addition, our results show that for dynamic systems such as barrier islands, sedimentation, and erosion dynamics can be also important determinants of species richness across taxa.

Looking at habitat diversity (or heterogeneity), a pattern emerging from our analyses is that taxa closely linked to primary producers (Lepidoptera, phytophagous Coleoptera) needed particularly high habitat diversity to reach high occurrence probabilities (Figure 4). Thus, trophic rank seems to affect species incidence, which has also been predicted by the trophic theory of island biogeography (Gravel, Massol, Canard, Mouillot, & Mouquet, 2011; Holt, 2009): while trophic theory predicts that the slope of species–area relationships increases with trophic rank (Holt, 2009), we may conclude from our study that the slope of the species‐heterogeneity relationship also increases with trophic rank. Higher trophic levels (that are often also more mobile; Holt, Polis, & Winemiller, 1996) have a higher probability of presence, even at low habitat diversity.

Our analyses of taxon multidiversity clearly could have benefitted from abundance data (rather than presence–absence data as in our case; Droege, Cyr, & Larivée, 1998; Richardson & Richards, 2008). This would have allowed to calculate “true” multidiversity indices (e.g., Allan et al., 2014), where species richness of each taxon could have been weighted by (maximum) abundance. As we had only incidence data, this was not possible here. Yet, the patterns observed are very strong and a change in these calculations would not have affected our conclusions.

Sorting species into taxonomic or functional groups is another potential point for discussion. Every sorting of species is arbitrary to some degree, and in the present manuscript some groupings could have been done differently (Scherber et al., 2010). Nevertheless, we believe that the groups chosen represent meaningful and interesting entities and will be useful especially from a conservation point of view.

Lastly, some critics could argue that we should have incorporated more explanatory variables into our models. Yet, we caution against this approach as our sample size (number of islands) was only *N* = 10, and we tried to restrict the range of variables entered as far as possible. Interesting extensions to our analyses could be to study trophic relationships among taxa, for example, between primary producers and herbivores. This could be a promising avenue for future research.

Disentangling the drivers of biodiversity in dynamic habitats is a major challenge, and the present study can only be a first step. In the future, experimental approaches and modeling should be employed to test for effects of area per se versus heterogeneity/habitat diversity (Triantis, Mylonas, Lika, & Vardinoyannis, 2003). We are in the process of installing experimental islands of known size and plant species composition (Balke et al., 2017) that will hopefully help to understand colonization and extinction processes, and hence community assembly processes, at multiple trophic levels in dynamic salt marsh ecosystems.

## CONFLICT OF INTEREST

None declared.

## AUTHOR CONTRIBUTIONS

HA, CS, and TT conceived the idea; RN and HA collected the data with additional material from collaborators; HA and CS analyzed the data; HA and CS jointly led the writing with assistance from TT and RN.

## DATA ACCESSIBILITY

The data supporting this study are available at https://doi.org/10.5061/dryad.j5j730p.
