# Supplementary material for: A barrier island perspective on species–area relationships
Source: Ecol Evol. 2018 Dec 8;8(24):12879–89. doi: 10.1002/ece3.4726 (PMC6308881; doi:10.1002/ece3.4726)
Supplement: Supplementary file 1 [file ECE3-8-12879-s001.docx]

**Supplementary Material**

**Table S1**

Lower triangle of the correlation matrix of explanatory variables. Shown are the Pearson correlation coefficients. Sedimentation, delta and erosion (with inverted sign) were log(x+1)-transformed. Variables never co-occurring in statistical models are shown in brackets.

|  | Area | Habitat diversity | Erosion | Sedimentation | Delta | Increase | CV |
| --- | --- | --- | --- | --- | --- | --- | --- |
| Area | 1.00 |  |  |  |  |  |  |
| Habitat diversity | (0.73) | 1.00 |  |  |  |  |  |
| Erosion | 0.48 | 0.29 | 1.00 |  |  |  |  |
| Sedimentation | 0.44 | 0.28 | (0.93) | 1.00 |  |  |  |
| Delta | 0.43 | 0.28 | (0.98) | (0.99) | 1.00 |  |  |
| Increase | 0.31 | 0.15 | 0.07 | -0.04 | 0.01 | 1.00 |  |
| CV | -0.29 | (-0.67) | -0.30 | -0.35 | -0.34 | (0.56) | 1.00 |

**Table S2**

Table showing the initial set of 23 models on species occurrence with pairwise combinations of explanatory variables included. Including all pairs would have resulted in 29 models (21 models containing two variables, plus 7 models including one variable, plus the null model). Including more than two variables per model would have been meaningless as sample size was only N=10 islands.

| **Model** | **Variable 1** | **Variable 2** |
| --- | --- | --- |
| 1 | Erosion | Island size |
| 2 | Erosion | Habitat diversity |
| 3 | Erosion | Growth in island size |
| 4 | Growth in island size | Difference in island size |
| 5 | Growth in island size | Sedimentation |
| 6 | Erosion | CV in island size |
| 7 | CV in island size | Difference in island size |
| 8 | CV in island size | Sedimentation |
| 9 | Island size | Sedimentation |
| 10 | Island size | Difference in island size |
| 11 | Habitat diversity | Sedimentation |
| 12 | Habitat diversity | Difference in island size |
| 13 | CV in island size | Island size |
| 14 | Growth in island size | Island size |
| 15 | Growth in island size | Habitat diversity |
| 16 | Difference in island size |  |
| 17 | Erosion |  |
| 18 | Sedimentation |  |
| 19 | Growth in island size |  |
| 20 | Island size |  |
| 21 | Habitat diversity |  |
| 22 | CV in island size |  |
| 23 | 1 |  |
